# Prognostic significance of peripheral blood inflammatory biomarkers (SII, PLR, NLR, LMR, SIRI, PIV, PNI) in lip cancer: NLR as an independent biomarker for survival outcomes

**DOI:** 10.3389/fonc.2025.1676824

**Published:** 2025-10-21

**Authors:** Zhilin Li, Wei An, Bin Wang, Zening Yan, Liangbin Gao, Jiaming Wang, Hongmei Yu, Shuxin Wen

**Affiliations:** ^1^ Department of Otolaryngology Head and Neck Surgery, Shanxi Bethune Hospital, Shanxi Academy of Medical Sciences, Third Hospital of Shanxi Medical University, Tongji Shanxi Hospital, Taiyuan, China; ^2^ Department of Head and Neck Surgery, Shanxi Provincial Cancer Hospital/Shanxi Hospital Affiliated to Cancer Hospital, Chinese Academy of Medical Sciences/Cancer Hospital Affiliated to Shanxi Medical University, Taiyuan, China; ^3^ Department of Oral and Maxillofacial Surgery, Shanxi Provincial People’s Hospital, The Fifth Clinical Hospital of Shanxi Medical University, Taiyuan, Shanxi, China; ^4^ Department of Stomatology, Tianjin Medical University General Hospital, Tianjin, China; ^5^ School of Stomatology, Shanxi Medical University, Taiyuan, China; ^6^ School of Public Health, Shanxi Medical University, Taiyuan, China

**Keywords:** lip cancer, peripheral blood inflammatory biomarker (PBIB), neutrophil-lymphocyte ratio (NLR), prognostic factors, survival outcomes

## Abstract

**Background:**

Lip cancer is a type of oral cancer with a different prognosis than that of other cancers. However, a lack of well-established understanding of the relationship between the peripheral blood inflammatory biomarker (PBIB) and prognosis in patients with lip cancer is evident. This study investigated the prognostic value of inflammatory markers and other unfavourable prognostic factors. It compares the systemic immune-inflammation index (SII), neutrophil-lymphocyte ratio (NLR), platelet-lymphocyte ratio (PLR), and lymphocyte-monocyte ratio (LMR), systemic inflammation response index (SIRI), pan-immune-inflammation value (PIV) and prognosis nutritional index (PNI) in patients with lip cancer.

**Materials and methods:**

This retrospective study included 122 patients with lip cancer. Clinical characteristics and hematological parameters were retrospectively obtained prior to treatment. SII, PLR, NLR, LMR, SIRI, PIV and PNI were calculated to analyze their effects on survival and recurrence further.

**Results:**

Receiver operating characteristics curve analysis demonstrated SII >534.286, PLR >146.528, NLR >2.134, LMR ≤4.000, SIRI >0.7100, PIV >211.930, PNI ≤51.900 were factors associated with increased mortality. Univariate analysis showed that these inflammatory parameters were associated with a lower survival rate. In multivariate analysis, NLR was identified as having a cumulative role in predicting overall survival (HR=5.885, 95% CI: 2.131–16.256, *P*<0.001).

**Conclusion:**

This study revealed that NLR is a promising blood biomarker in patients with lip cancer. The predictive power of other PBIBs, albeit showing a trend towards significance, were not statistically significant, possibly due to the limited number of cases. The clinical applicability of other hematological indicators requires further study.

## 1 Introduction

Cancer is the leading cause of death in both developed and developing countries. Oral cavity and pharynx cancer is the 6^th^ most common malignant tumor in the world. Approximately 700,000 newly diagnosed cases and 350,000 deaths occur annually worldwide. A National Cancer Institute study showed that the 5-year relative mortality of oral and pharyngeal cancer exceeds 30% (1). However, cancer in the front of the mouth, such as lip cancer, has a better prognosis with lower mortality (<15%) (2) than cancers in other areas of the oral cavity and pharynx do, such as those in the tongue (68.8%) (3), floor of the mouth (>60%) (4), laryngeal (61%), oropharyngeal (41%) and hypopharyngeal (25%) (5).

Inflammation is a pivotal factor in tumors, and growing evidence indicates that it can regulate the tumor microenvironment and immune reaction to accelerate tumor proliferation, progression, and lymph node metastasis (6). Peripheral blood inflammatory biomarkers (PBIBs), including the neutrophil-lymphocyte ratio (NLR), lymphocyte-monocyte ratio (LMR), platelet-lymphocyte ratio (PLR), and systemic immune-inflammation index (SII), have been reported in head and neck cancers. In recent years, many new biomarker such as systemic inflammation response index (SIRI), pan-immune-inflammation value (PIV) and prognosis nutritional index (PNI) have been proven to affect survival in various cancer type (7). All these peripheral blood inflammatory biomarkers primarily encompass ratios between neutrophils, lymphocytes, platelets, monocytes, and serum albumin levels. Neutrophils, one of the primary effector cells in inflammatory responses, release pro-inflammatory cytokines and chemokines that promote tumor invasion and metastasis (8). Lymphocytes play an important role in the immune system and are capable of recognizing and eliminating tumor cells. Platelets are essential elements of innate and adaptive immune responses, and abnormal platelets also contribute to tumor metastasis and poor prognosis (9). Monocytes have a strong inhibitory effect on a variety of antitumor immune cells. In many tumors, such as prostate cancer (10), breast cancer (11), and colorectal cancer (12), a high number of monocytes is associated with poor prognosis.

Several meta-analyses of oral squamous cell carcinoma (OSCC) have confirmed that inflammatory biomarkers such as the NLR and PNI serve as reliable predictors of cancer prognosis (7, 13). Beyond OSCC, a growing body of evidence underscores the broad prognostic utility of these markers across various head and neck malignancies. For instance, in malignant salivary gland tumors, the systemic immune-inflammation index (SII) and systemic inflammation response index (SIRI) have been shown to independently predict overall survival, with their combination offering high prognostic accuracy (AUC=0.884) and significantly stratifying 5-year survival outcomes (14). Moreover, the clinical relevance of these biomarkers extends beyond survival prognostication to include the detection of occult lymph node metastases. In early-stage oral cavity carcinomas (T1-T2/N0), inflammatory markers such as PLR, NLR, and SII demonstrated high diagnostic accuracy in identifying occult neck metastases, thereby supporting preoperative decision-making regarding neck management. However, while these studies affirm the value of systemic inflammation indices in sites such as the oral cavity and salivary glands, their predictive power for long-term survival and recurrence specifically in lip cancer remains insufficiently elucidated (15). The current study therefore aimed to comprehensively evaluate the prognostic significance of an extended panel of inflammatory and nutritional marker, including SII, NLR, PLR, LMR, SIRI, PIV, and PNI, in a well-defined cohort of lip cancer patients.

## 2 Materials and methods

### 2.1 Patients and methods

A retrospective analysis was conducted on patients with lip cancer treated at Shanxi Province Cancer Hospital between September 1999 and August 2020. The inclusion criteria for this study were as follows: (1) Histopathologically confirmed carcinoma of the lip (Squamous cell carcinoma, basal cell carcinoma, verrucous carcinoma, etc.); (2) Underwent curative-intent surgical resection and/or radiotherapy as the primary treatment (wide local excision, with or without neck dissection, and/or reconstructive surgery); (3) Availability of peripheral blood specimens collected within one week prior to any treatment; (4) Availability of complete clinical information and follow-up data. To ensure a comprehensive analysis of the biomarkers’ prognostic value across disease severities, patients across all T and N stages, including those with clinically or pathologically confirmed neck lymph node metastases, were included. Exclusion criteria: (1) Presence of synchronous or metachronous malignancies in other organs; (2) Presence of preoperative active infection or systemic inflammatory conditions (inflammatory bowel disease, autoimmune disorders) that could confound the inflammatory biomarker levels; (3) History of prior radiotherapy (RT) or chemotherapy (CMT) for any condition in the head and neck region; (3) Patients with incomplete medical records or lost to follow-up.

In total, 122 patients with lip cancer met the criteria and were enrolled in the present study (89 men and 33 women; median age 64 years; interquartile range: 52.00-73.25 years). These patients were classified and staged according to the Union for International Cancer Control tumor-node-metastasis (TNM) classification for head and neck cancer.

This retrospective study was approved by the Ethics Committee of the authors’ affiliated institution (approval number: KY2024048). The requirement of informed consent was waived.

### 2.2 Data collection

The following clinical characteristics were retrospectively reviewed using inpatient records: sex, age, BMI, tumor size, tumor location, surgical treatment, neck dissection, radiotherapy, tumor stage, pathological type, prognosis, lymph node metastasis, death, and recurrence.

All blood specimens were acquired from patients within 7 days of treatment. Peripheral blood cell counts were determined and classified, including neutrophils, monocytes, lymphocytes, and platelets. Inflammatory indices were calculated based on previously established methods (16–18): SII = platelets × neutrophils/lymphocytes, NLR = neutrophil count/lymphocyte count, PLR =platelet count/lymphocyte count, LMR = lymphocyte count/monocyte count, SIRI = (neutrophil count × monocyte count)/lymphocyte count, PIV = (platelet count × neutrophil count × monocytes count)/lymphocyte count, serum albumin + 5 × lymphocyte count was used to determine the PNI.

Clinical staging was determined in accordance with the AJCC Cancer Staging Manual (8th Edition). All patients underwent a standardized diagnostic workup, which included a comprehensive physical examination, high-resolution ultrasonography of the neck to assess lymph node status, and computed tomography (CT) or magnetic resonance imaging (MRI) to evaluate the local extent of the primary tumor and rule out distant metastasis. The final pathological stage (pTNM) was assigned for surgically treated patients based on histopathological examination of the resection specimens.

After primary treatment, patients were regularly followed up according to our institutional protocol. Follow-up examinations were scheduled every 3 months for the first two years, every 6 months for the next three years, and annually thereafter. Each follow-up visit included a thorough physical examination of the head and neck region and ultrasonography of the neck. Additional imaging (CT, MRI, or PET-CT) was performed if recurrence or metastasis was clinically suspected. Disease-free survival (DFS) was defined as the time from diagnosis to the first event of disease recurrence or death from any cause. Disease recurrence was defined as the confirmed reappearance of the tumor following primary treatment, encompassing local (at the primary site), regional (in cervical lymph nodes), or distant metastasis.

### 2.3 Statistical analysis

Statistical analyses were performed using SPSS software (version 26.0; IBM Corp., Armonk, NY, USA). The receiver operating characteristic (ROC) curve was used to identify and determine the optimal cutoff values of the SII, NLR, PLR, LMR SIRI, PIV and PNI. Using the maximum area under the curve (AUC) method, we systematically evaluated the predictive performance of each biomarker at different threshold levels for 5-year overall survival. The optimal cutoff points were selected based on thresholds yielding the highest AUC values, thereby maximizing their prognostic discrimination capability.

The Mann-Whitney U test and the chi-square test were used to analyze the differences between inflammatory indices and the prognosis of lip cancer, respectively. Univariate analyses and Cox proportional regression models were used to estimate the predictive value of the variables. Kaplan-Meier curves were generated to visualize the prognosis of the variables. The log-rank test was used to analyze survival differences between the groups and to draw a survival curve. All statistical analyses were performed using bilateral *P*<0.05, which indicated a statistically significant difference.

## 3 Results

### 3.1 Patient characteristics

Detailed characteristics of the 122 patients are shown in **Table 1**. In short, the patients included 89 (73.00%) males and 33 (27.00%) females aged 17–90 years. The mean age at diagnosis was 64 (52.00, 73.25) years. 36.89% (45/122) of participants reported having a smoking history, while 11.48% (14/122) indicated a history of alcohol consumption. In the current study, most lesions were located in the lower lip (73.00%), while 33 (27.00%) were located in the upper lip, with a mean tumor size of 2.36 ± 1.14cm. In this study, the treatment modalities for lip cancer consisted of surgery alone (97 patients, 79.51%), surgery combined with radiotherapy (15 patients, 12.29%), and radiotherapy alone (10 patients, 8.20%). No patients underwent chemotherapy as part of their treatment regimen. Most of patients (84.40%) were diagnosed with squamous cell carcinoma, 15.60% of patients were other clinicopathologic results. 64 (52.50%) patients were at TNM stage I, 43 (35.20%) were at stage II. Neck dissection was performed in 45 patients (36.90%), among whom 13 (10.70% of total patients) had lymph node metastasis. The complete blood count analysis revealed the following median values with interquartile ranges: neutrophils 3.74 (2.95-4.67) × 10^9^/L, monocytes 1.95 (1.56-2.54) × 10^9^/L, and lymphocytes 0.39 (0.32-0.48) × 10^9^/L Platelets averaged 217.29 ± 67.98 × 10^9^/L.

**Table 1:** Baseline characteristics of 122 patients.

Patient characteristics	N = 122 (%)
Age (median, inter-quartile range)	64 (52.00, 73.25)
Gender	
Male	89 (73.00%)
Female	33 (27.00%)
BMI (median, inter-quartile range)	22.72 (20.52, 25.47)
Smoking	
Yes	45 (36.89%)
No	77 (63.11%)
Alcohol	
Yes	14 (11.48%)
No	108(88.52%)
Tumor Size (median, inter-quartile range)	2.00 (1.50, 3.00)
Location of the tumor	
Upper lip	33 (27.00%)
Lower lip	89 (73.00%)
Treatment	
Surgery	97(79.51%)
Surgery+Radiotherapy	15(12.29%)
Radiotherapy	10(8.20%)
TNM Stage	
I	64 (52.50%)
II	43 (35.20%)
III	12 (9.80%)
IV	3 (2.50%)
Squamous cell carcinoma	
No	19 (15.60%)
Yes	103 (84.40%)
Neck dissection	
No	77 (63.10%)
Yes	45 (36.90%)
Lymph node metastasis	
No	109 (89.30%)
Yes	13 (10.70%)
Recurrence	
Yes	16 (13.11%)
No	106 (86.89%)
Death	
Yes	20 (16.39%)
No	102 (83.61%)
Neutrophil count (median, inter-quartile range) (10^9^/L)	3.74 (2.95, 4.67)
Lymphocyte count (median, inter-quartile range) (10^9^/L)	1.95 (1.56, 2.54)
Monocyte count (median, inter-quartile range) (10^9^/L)	0.39 (0.32, 0.48)
Platelet count (Mean ± SD) (10^9^/L)	217.29±67.98

BMI, Body Mass Index; TNM Stage, Tumor-Node-Metastasis Stage; SD, Standard Deviation.

### 3.2 Prognostic performance of inflammatory biomarkers

**Figure 1:**
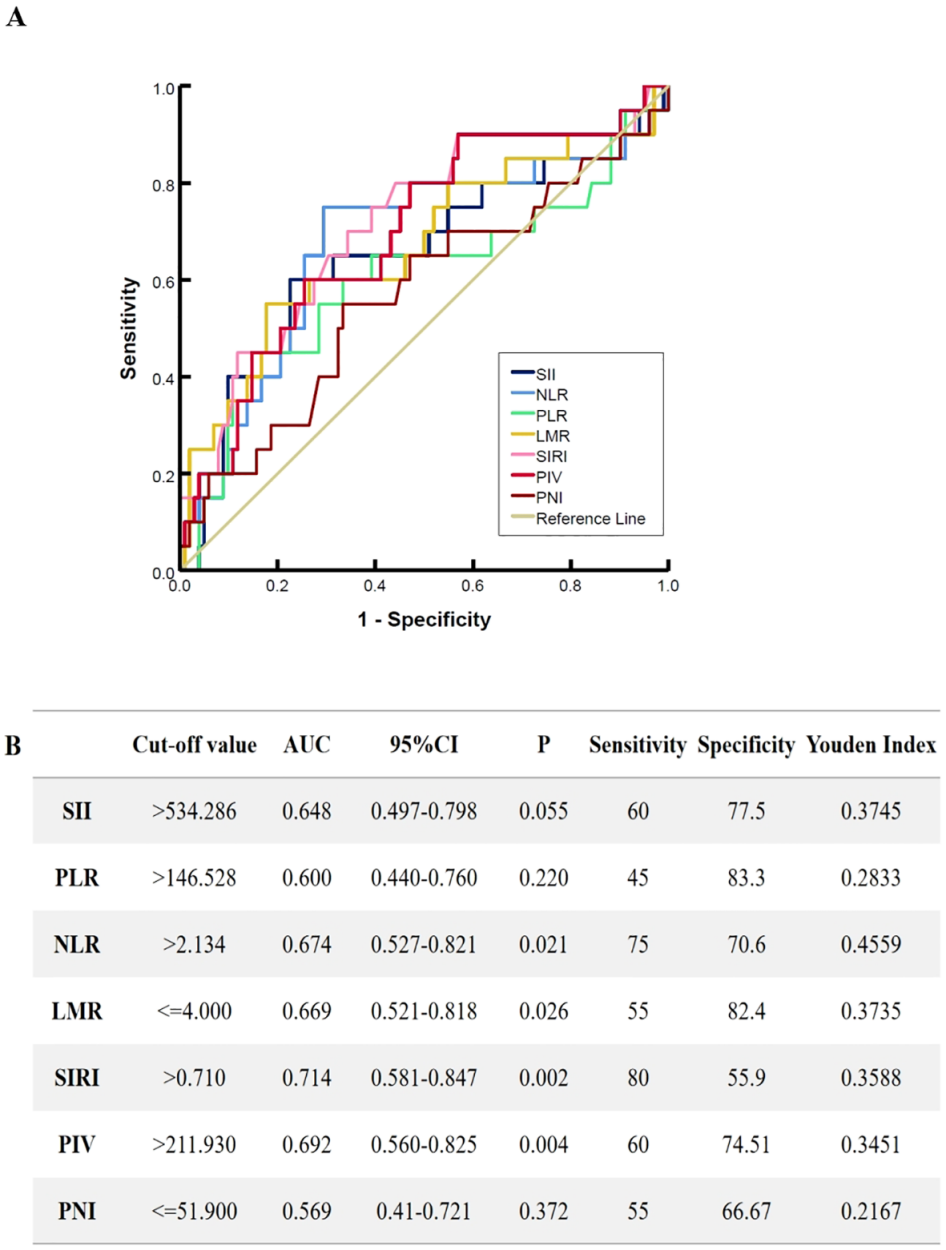
The receiver operating characteristic curves for pretreatment inflammation markers. **(A)** the ROC characteristic of SII, NLR, LMR, PLR, SIRI, PIV and PNI, **(B)** The selection of the optimal cutoff values and predictive power of inflammation markers on patient. SII, systemic immune/inflammatory response index calculated by multiplying platelets by neutrophils and divided by lymphocytes; NLR, neutrophil/lymphocyte ratio; LMR, lymphocyte/monocyte ratio; PLR, platelet/lymphocyte ratio; SIRI, systemic inflammation response index; PIV, pan-immune-inflammation value; PNI, prognosis nutritional index.

Using the SII, NLR, LMR, PLR, SIRI, PIV, and PNI as test variables, ROC curves were used to calculate and identify the optimal cutoff values of the inflammatory markers for overall survival. The optimal cutoff values were as follows: SII=534.286; PLR=146.528; NLR=2.134; LMR=4.000; SIRI=0.710; PIV=211.930 and PNI=51.900. **Figure 1** shows the ROC curves of the pretreatment inflammation indexes and nutritional index.

To elucidate the significance of these peripheral blood related indexes in the prognosis of lip cancer, all patients were divided into two groups based on the optimal cutoff points for further analysis (low and high values). The correlations among the SII, NLR, PLR, LMR, SIRI, PIV, PNI and clinicopathological characteristics of the patients were investigated (**Table 2**). In this study, the NLR and SIRI groups analysis indicated no significant baseline differences between the high and low cutoff values groups. The high SII group was significantly correlated with a larger tumor size (*P* =0.006), and significant differences in TNM stage were observed between the higher PLR group and the lower group (*P* =0.005). Differences of tumor size (*P* =0.001) were obvious between different PIV groups. The Age (*P <*0.003) and different treatment approaches (*P <*0.001) differed significantly between PNI groups.

**Table 2:** Correlation between hematological parameters and clinicopathological characteristics of lip cancer patients.

(1)												
Characteristics	SII		P	NLR		P	LMR		P	PLR		P
	Low	High		Low	High		Low	High		Low	High	
	≤534.286	>534.286		≤2.134	>2.134		≤4.000	>4.000		≤146.528	>146.528	
Age	64.5 (52.8, 72.3)	63.5 (49.5, 74.0)	0.647	63.0 (50.0, 72.0)	70.0 (56.0, 75.0)	0.065	70.5 (55.5, 75.0)	63.0 (50.5, 72.0)	0.057	63.0 (51.3, 72.0)	65.5 (58.3, 77.5)	0.095
Gender			0.742			0.942			0.212			0.630
Male	62	27		56	33		23	66		71	18	
Female	24	9		21	12		5	28		25	8	
BMI	22.5 (20.4, 26.3)	22.9 (21.4, 25.3)	0.703	22.8 (20.8, 25.9)	22.7 (19.8, 25.3)	0.712	22.5 (20.3, 24.7)	22.8 (20.7, 26.3)	0.377	22.8 (20.6, 26.3)	22.5 (19.5, 25.0)	0.331
Smoking			0.599			0.876			0.456			0.466
No	53	24		49	28		16	61		59	18	
Yes	33	12		28	17		12	33		37	8	
Alcohol			0.588			0.923			0.227			0.991
No	77	31		68	40		23	85		85	23	
Yes	9	5		9	5		5	9		11	3	
Tumor size	2.0 (1.5, 3.0)	2.8 (2.0, 4.0)	0.006*	2.0 (1.5, 3.0)	2.0 (2.0, 3.0)	0.596	2.5 (2.0, 4.0)	2.0 (1.5, 3.0)	0.06	2.0 (1.5, 3.0)	2.5 (2.0, 3.0)	0.114
Tumor location			0.907			0.106			0.781			0.987
Upper lip	23	10		17	16		7	26		26	7	
Lower lip	63	26		60	29		21	68		70	19	
Treatment			0.332			0.077			0.105			0.122
Surgery	70	27		64	33		20	77		80	17	
Surgery +Radiotherapy	11	4		10	5		3	12		10	5	
Radiotherapy	5	5		3	7		5	5		6	4	
Neck dissection			0.599			0.586			0.554			0.787
No	53	24		50	27		19	58		60	17	
Yes	33	12		27	18		9	36		36	9	
TNM stage			0.165			0.135			0.253			0.005*
1	50	14		40	24		12	52		54	10	
2	27	16		29	14		11	32		32	11	
3	8	4		8	4		3	9		10	2	
4	1	2		0	3		2	1		0	3	
Squamous cell carcinoma			0.379			0.997			0.419			0.212
No	15	4		12	7		3	16		17	2	
Yes	71	32		65	38		25	78		79	24	
Lymph node metastasis			0.454			0.464			0.991			0.378
No	78	31		70	39		25	84		87	22	
Yes	8	5		7	6		3	10		9	4	
(2)													
Characteristics	SIRI		P	PIV		P	PNI		P			
	Low	High		Low	High		Low	High				
	≤0.71	>0.71		≤211.93	>211.93		≤51.90	>51.90				
Age	63.0 (51.5,72.0)	65.0 (52.5,74.5)	0.684	64.5 (52.0,72.8)	63.5 (52.5,74.0)	0.788	68.0 (60.0,76.5)	62.0 (48.5,71.5)	0.003*			
Gender			0.541			0.149			0.942			
Male	43	46		58	31		33	56				
Female	18	15		26	7		12	21				
BMI	22.5 (20.8,26.6)	22.8 (20.3,25.2)	0.678	22.5 (20.6,26.5)	22.9 (20.3,25.1)	0.868	22.2 (20.1,25.0)	23.1 (20.7,26.5)	0.301			
Smoking			0.573			0.422			0.312			
No	40	37		55	22		31	46				
Yes	21	24		29	16		14	31				
Alcohol			0.570			0.315			0.280			
No	55	53		76	32		38	70				
Yes	6	8		8	6		7	7				
Tumor size	2.0 (1.5,3.0)	2.0 (2.0,3.3)	0.153	2.0 (1.5,3.0)	3.0 (2.0,4.0)	0.001*	2.0 (2.0,3.0)	2.0 (1.5,3.0)	0.675			
Tumor location			0.839			0.902			0.727			
Upper lip	16	17		23	10		13	20				
Lower lip	45	44		61	28		32	57				
Treatment			0.788			0.393			<0.001*			
Surgery	68	29		68	29		29	68				
Surgery +Radiotherapy	11	4		11	4		6	9				
Radiotherapy	5	5		5	5		10	0				
TNM stage			0.307			0.070			0.597			
1	34	30		50	14		24	40				
2	22	21		27	16		16	27				
3	5	7		6	6		3	9				
4	0	3		1	2		2	1				
Squamous cell carcinoma			0.301			0.301			0.608			
No	15	4		15	4		8	11				
Yes	69	34		69	34		37	66				
Neck dissection			0.348			0.414			0.162			
No	41	36		51	26		32	45				
Yes	20	25		33	12		13	32				
Lymph node metastasis			0.216			0.216			0.275			
No	77	32		77	32		42	67				
Yes	7	6		7	6		3	10				

SII, systemic immune-inflammation index; NLR, neutrophil-lymphocyte ratio; LMR, lymphocyte-monocyte ratio; PLR, platelet-lymphocyte ratio; SIRI, systemic inflammation response index; PIV, pan-immune-inflammation value; PNI, prognosis nutritional index.

*, p<0.05.

When inflammation indices and other clinicopathological characteristics were analyzed, there were no significant differences in the location of lip cancer, neck dissection, lymph node status, or pathological type. Both groups across the seven inflammation indices were associated with neutrophils, lymphocytes (*P <*0.05) (**Table 3**), LMR, SIRI, PIV and PNI groups were significantly correlated with monocytes count; SII, PLR, SIRI, PIV and PNI groups were significantly correlated with Platelet count.

**Table 3:** Correlation between biomarkers for hematological parameter and haemocytes of lip cancer patients.

Characteristics		Neutrophils (10^9^/L)	P	Lymphocyte (10^9^/L)	P	Monocytes (10^9^/L)	P	Platelet (10^9^/L)	P
SII	Low	3.23 (2.63, 3.83)	<0.001*	2.09 (1.72, 2.68)	<0.001*	0.36 (0.32, 0.47)	0.292	198.13 ± 55.38	<0.001*
	High	5.06 (4.37, 6.23)		1.56 (1.24, 2.11)		0.41 (0.31, 0.55)		263.06 ± 73.97	
NLR	Low	3.22 (2.63, 3.97)	<0.001*	2.26 (1.91, 2.85)	<0.001*	0.38 (0.32, 0.48)	0.834	213.48 ± 63.30	0.421
	High	4.46 (3.79, 6.01)		1.55 (1.21, 1.72)		0.39 (0.31, 0.50)		223.80 ± 75.62	
LMR	Low	4.18 (3.60, 5.21)	0.010*	1.57 (1.20, 1.75)	<0.001*	0.45 (0.39, 0.59)	<0.001*	201.79 ± 73.50	0.170
	High	3.37 (2.79, 4.46)		2.13 (1.72, 2.79)		0.35 (0.29, 0.46)		221.90 ± 65.96	
PLR	Low	3.55 (2.84, 4.47)	0.035*	2.13 (1.73, 2.77)	<0.001*	0.40 (0.32, 0.51)	0.09	205.55 ± 62.31	<0.001*
	High	4.35 (3.34, 5.33)		1.39 (1.00, 1.65)		0.35 (0.29, 0.43)		260.62 ± 71.63	
PIV	Low	1.59(1.16,2.03)	<0.001*	5.89(4.72,7.26)	<0.001*	0.17(0.14,0.21)	<0.001*	198.95 ± 55.38	<0.001*
	High	2.74(2.17,4.03)		3.95(2.46,4.83)		0.25(0.21,0.41)		257.84 ± 76.02	
PNI	Low	2.33(1.73,3.84)	<0.001*	4.6(3.42,5.79)	<0.001*	0.22(0.17,0.29)	<0.001*	192.76 ± 59.96	0.002*
	High	1.66(1.22,2.11)		5.78(4.55,7.23)		0.17(0.14,0.22)		231.62 ± 68.64	
SIRI	Low	1.40(1.06,1.7)	<0.001*	6.37(5.31,7.66)	<0.001*	0.16(0.13,0.19)	<0.001*	203.90 ± 57.43	0.029*
	High	2.37(2.02,3.69)		4.23(3.42,5.13)		0.24(0.2,0.29)		230.67 ± 75.21	

SII, systemic immune-inflammation index; NLR, neutrophil-lymphocyte ratio; LMR, lymphocyte-monocyte ratio; PLR, platelet-lymphocyte ratio; SIRI, systemic inflammation response index; PIV, pan-immune-inflammation value; PNI, prognosis nutritional index. *, p<0.05.

### 3.3 Survival analysis

**Figure 2:**
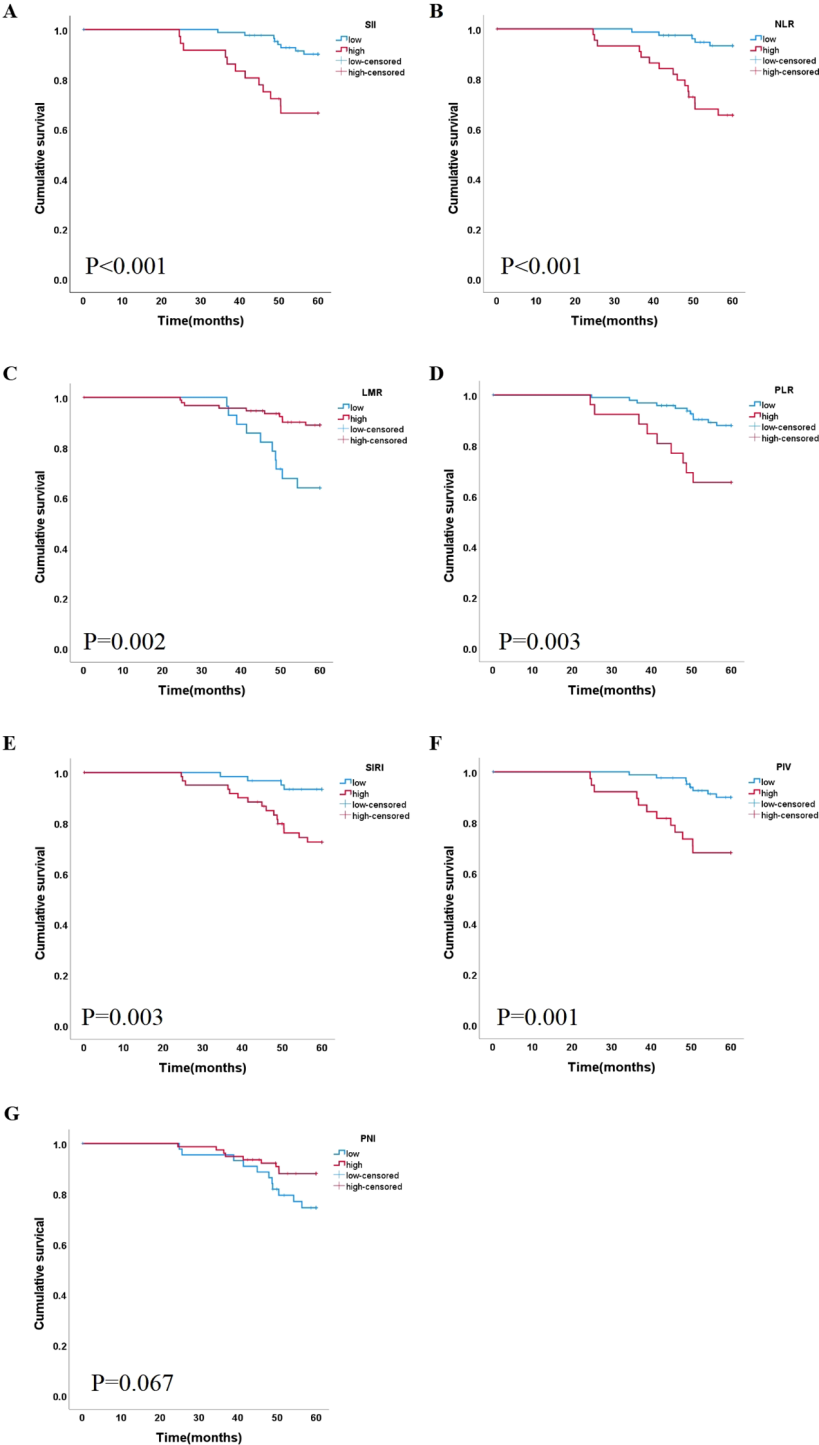
Correlation between the level of 7 indicators and lip cancer survival. Kaplan-Meier curves based on inflammation markers **(A)** SII, **(B)** NLR **(C)** LMR, **(D)** PLR, **(E)** SIRI, **(F)** PIV and **(G)** PNI SII, systemic immune/inflammatory response index calculated by multiplying platelets by neutrophils and divided by lymphocytes; NLR, neutrophil/lymphocyte ratio; LMR, lymphocyte/monocyte ratio; PLR, platelet/lymphocyte ratio; SIRI, systemic inflammation response index; PIV, pan-immune-inflammation value; PNI, prognosis nutritional index.

A Kaplan–Meier curve was used in the survival analyses of the different inflammation indices. **Figure 2** shows that the cumulative survival rate in patients with a lower SII, NLR, PLR, SIRI, and PIV was better than that in other patients (*P*<0.050). In the group of NLR ≥2.134, survival was negatively correlated with NLR values. The high LMR group exhibited a superior survival rate compared to the low LMR group (*P*<0.050), Only the groups of PNI had no significant difference in cumulative survival rate.

During follow-up, 16 (13.11%) patients had disease recurrence, and 20 (16.39%) patients died. Descriptive data are presented in **Table 1**. These variables were identified as independent predictors using univariate and multivariate analyses.

Univariate logistic regression analysis showed that age (HR=1.044, 95% CI: 1.007-1.083, *P*=0.020), TNM stage (HR=1.921, 95% CI: 1.189-3.104, *P*=0.008), Surgery (HR=0.305, 95% CI: 0.102-0.912, *P*=0.034) and lymph node metastases (HR=4.634, 95% CI: 1.771-12.125, *P*=0.002) were all associated with lower survival rates. The inflammation indices in our study, SII (HR=4.226, 95% CI: 1.726-10.347, *P*=0.002), NLR (HR=6.209, 95% CI: 2.254-17.101, *P*<0.001), and PLR (HR=3.480, 95% CI: 1.441-8.405, *P*=0.006), LMR (HR=0.269, 95% CI: 0.112-0.647, *P* =0.003), SIRI (HR=4.633, 95% CI: 1.548-13.866, *P*=0.006), PIV (HR=3.902, 95% CI: 1.594-9.554, *P*=0.003),were all factors associated with lower overall survival (**Table 4**).

**Table 4:** Univariate and multivariate analysis for disease-free survival in lip cancer patients.

Characteristics	Univariate Cox			Multivariate Cox		
	HR	95%CI	P	HR	95%CI	P
Gender	0.618	0.207–1.849	0.389			
Age (years)	1.044	1.007–1.083	0.020*	1.038	1.002-1.077	0.040*
BMI	0.895	0.778–1.029	0.118			
Smoking	0.925	0.369-2.317	0.867			
Alcohol	1.506	0.441-5.144	0.514			
Tumor location	0.670	0.267–1.679	0.393			
Tumor size	1.341	0.956–1.879	0.089			
Squamous cell carcinoma	3.653	0.489-27.288	0.207			
Surgery	0.305	0.102-0.912	0.034*			
Radiotherapy	1.255	0.456-3.453	0.660			
Treatment						
Surgery (reference)	1	–	0.124			
Surgery +Radiotherapy	0.387	0.051-2.928	0.358			
Radiotherapy	2.616	0.868-7.884	0.088			
TNM stage	1.921	1.189–3.104	0.008*			
Pathological classification	3.653	0.489–27.288	0.207			
Neck dissection	0.943	0.376–2.365	0.901			
Lymph node metastasis	4.634	1.771–12.125	0.002*	4.649	1.749-12.357	0.002*
SII	4.226	1.726–10.347	0.002*			
NLR	6.209	2.254–17.101	<0.001*	5.885	2.131-16.256	<0.001*
LMR	0.269	0.112–0.647	0.003*			
PLR	3.480	1.441–8.405	0.006*			
SIRI	4.633	1.548-13.866	0.006*			
PIV	3.902	1.594-9.554	0.003*			
PNI	0.448	0.186-1.082	0.074			

HR, Hazard Ratio; 95%CI, 95% Confidence Interval; TNM, Tumor-Node-Metastasis; SII, systemic immune-inflammation index; NLR, neutrophil-lymphocyte ratio; LMR, lymphocyte-monocyte ratio; PLR, platelet-lymphocyte ratio; SIRI, systemic inflammation response index; PIV, pan-immune-inflammation value; PNI, prognosis nutritional index. *, p<0.05.

A multivariate Cox regression analysis was performed to assess whether these parameters were independent predictors of overall survival (**Table 4**). The Age (HR=1.038, 95% CI: 1.002-1.077, *P*=0.040), lymph node metastases (HR=4.649, 95% CI: 1.749-12.357, *P*=0.002), and NLR (HR=5.324, 95% CI: 1.928-14.701, *P*<0.001) were shown to have a cumulative role in predicting overall survival. Furthermore, in the analysis of disease recurrence, both surgery (HR=0.183, 95% CI: 0.048-0.690, *P* =0.012) and lymph node metastases (HR=4.492, 95% CI: 1.347-14.977, *P* =0.014) were identified as significant factors. However, none of the inflammatory indices showed a significant association with recurrence in the multivariate analysis (**Table 5**).

**Table 5:** Univariate and multivariate analysis for disease recurrence in lip cancer patients.

Characteristics	Univariate Cox			Multivariate Cox		
	HR	95%CI	P	HR	95%CI	P
Gender	0.646	0.182–2.288	0.498			
Age (years)	1.004	0.968–1.042	0.819			
BMI	0.871	0.739–1.026	0.099			
Smoking	0.242	0.055-1.074	0.062			
Alcohol	1.713	0.589-4.979	0.323			
Tumor location	1.035	0.329–3.250	0.953			
Tumor size	1.102	0.714–1.702	0.660			
Squamous cell carcinoma	1.219	0.275-5.401	0.795			
Surgery	0.247	0.070-0.878	0.031*	0.183	0.048-0.690	0.012*
Radiotherapy	2.966	1.055-8.336	0.039*			
Treatment						
Surgery (reference)	1	–	0.097			
Surgery +Radiotherapy	2.432	0.658-8.988	0.183			
Radiotherapy	3.749	1.014-13.858	0.048*			
TNM stage	1.394	0.740-2.626	0.304			
Pathological classification	1.219	0.275–5.401	0.795			
Neck dissection	0.868	0.297–2.539	0.796			
Lymph node metastasis	3.480	1.104–10.968	0.033*	4.492	1.347–14.977	0.014*
SII	1.205	0.412–3.528	0.733			
NLR	0.877	0.300–2.566	0.811			
LMR	0.821	0.261–2.579	0.735			
PLR	1.375	0.438–4.319	0.585			
SIRI	0.669	0.238-1.879	0.445			
PIV	1.090	0.372-3.189	0.875			
PNI	0.472	0.171-1.301	0.147			

HR, Hazard Ratio; 95%CI, 95% Confidence Interval; BMI, Body Mass Index; TNM, tumor-node-metastasis; SII, systemic immune-inflammation index; NLR, neutrophil-lymphocyte ratio; LMR, lymphocyte-monocyte ratio; SIRI, systemic inflammation response index; PIV, pan-immune-inflammation value; PNI, prognosis nutritional index. *, p<0.05.

## 4 Discussion

Peripheral blood inflammatory biomarkers have demonstrated the prognostic significance in different diseases. Elevated inflammatory responses in the tumor microenvironment consistently correlating with poorer clinical outcomes (19). These biomarkers not only show significant associations with patient prognosis but also serve as independent prognostic factors in multiple cancer type (20), including lung cancer, renal cell carcinoma (21), and liver cancer (22). Inflammation indices have been established as predictors of carcinogenesis and disease progression in head and neck malignancies. However, most prior studies focused on the larynx, tongue, and oral cavity, our study presents an extensive analysis of systemic indices and their link to unfavourable prognosis in lip cancer, a topic not previously reported. The findings reveal correlations between peripheral blood inflammatory markers and both poor survival outcomes and disease recurrence in lip cancer patients. Furthermore, we systematically evaluate composite biomarkers including SIRI, PIV and PNI to elucidate the synergistic impacts of immune dysregulation, systemic inflammation, and nutritional status on disease progression. This multidimensional analysis provides novel insights into the correlation between host inflammatory responses and lip cancer outcomes.

The indices SII, NLR, PLR, LMR, SIRI, PIV and PNI were associated with poor overall survival in the univariate analysis. Kaplan-Meier curves in our study demonstrated that SII, NLR, PLR, SIRI, and PIV were positively correlated with poor prognosis in lip cancer, while LMR showed a negative correlation. Blood inflammatory biomarkers have better prognostic value than nutrition-related index. These biomarkers are closely linked to the inflammatory-immune imbalance within TME. Low LMR or elevated NLR reflects lymphocytopenia, indicating impaired immune surveillance that leads to insufficient CD8^+^ T cell responses against tumor-specific antigens. Additionally, a high MLR may be associated with monocyte differentiation into M2-polarized macrophages, which suppress local immune activity through TGF-β secretion and promote the maintenance of cancer stem cell-like phenotypes. Lip cancer likely sustains a low-grade inflammatory state in the TME, resulting in persistently elevated PBIBs. This chronic inflammation further activates pro-survival genes via the NF-κB signaling pathway, exacerbating therapeutic resistance (23). Collectively, these mechanisms elucidate the independent prognostic value of PBIBs for survival outcomes in lip cancer and provide a theoretical foundation for future precision therapies targeting inflammatory pathways.

But, in the multivariate analysis, only NLR was significantly correlated with poor prognosis (*P <*0.001). This study shows that NLR has independent prognostic value in lip cancer, which is consistent with previous research on other head and neck cancers (24). Most of these studies have shown that a high NLR in cancer patients is associated with poorer clinical outcomes in cancer patients (25, 26). This study demonstrated that the optimal cutoff value of NLR was 2.134, which was lower than that in many studies on head and neck cancer (27). This discrepancy may reflect distinct pathophysiological characteristics of lip cancer, including its unique ultraviolet-induced inflammatory patterns and fundamentally different tumor immune microenvironment (26, 28).

Elevated NLR signifies neutrophilia (pro-inflammatory state) and lymphopenia (immunosuppression), mirroring a tumor microenvironment (TME) skewed toward tumor progression. Neutrophils, the most abundant leukocyte in the innate immune system, originate from myeloid progenitor cells in the bone marrow, have a short lifespan but are highly active (8). The homeostasis of neutrophil numbers is finely regulated by positive and negative feedback signals. In cancer, this balance can be disrupted, the number of neutrophils in peripheral blood is often elevated. Within the tumor microenvironment, neutrophils release ROS/RNS, which can damage DNA bases and promote tumor growth. They also secrete various cytokines (e.g., OSM, TGF-β) and chemokines (e.g., IL17, CXCR2 ligands) in response to different stimuli, thereby driving tumor cell proliferation, angiogenesis, and immunosuppression (29, 30).

During the metastatic process, neutrophils promote tumor progression through stage-specific mechanisms: in the early stage, they secrete proteases to enhance tumor cell invasive capabilities; in the intermediate stage, they support tumor cell survival and immune evasion; and in the late stage, they accumulate in the pre-metastatic niche and release factors to stimulate tumor cell proliferation and angiogenesis (29). Elevated neutrophil counts correlate with worse prognosis, forming the basis of NLR. Lymphocytes, conversely, mediate antitumor immunity by inhibiting tumor proliferation and migration. Reduced lymphocyte counts impair immune surveillance, further worsening outcomes (31).

Furthermore, we found that all the inflammatory markers were associated with neutrophils. The effect of neutrophils on various inflammatory indicators and their important roles in prognosis have also been demonstrated. Therefore, the effect of neutrophils on oral cancer, especially lip cancer, may be an issue that we need to explore further.

However, lip cancer is often caused by UV exposure, leading to unique molecular and inflammatory features compared to HNCs linked to HPV or tobacco use (32). This may explain why other PBIBs, like SII and PIV, are less predictive in lip cancer. In lip cancer, UV-induced neutrophil infiltration and oxidative stress boost NLR’s prognostic importance, while in HPV-negative HNCs or tobacco - related cases, PD-L1 driven T-cell exhaustion or platelet activation makes other PBIBs more clinically relevant. Previous research on the association of these indicators with OSCC prognosis has been inconclusive, and our findings revealed no significant statistical difference (13, 33–35). Therefore, whether we can use all these inflammatory indicators (except NLR) to build a prediction model for cancer requires further research.

Therefore, if the above indices can be reasonably used to increase the sensitivity and specificity of the prediction model, tumor prognosis can be better evaluated. We hypothesized that the combined inflammatory index might be helpful in determining adverse risks in patients with lip cancer and has an important role in predicting cancer-free survival.

In this study, we also analyzed the association between disease recurrence and inflammation indices and found no significant differences between the two groups. Lip cancer recurrence was only affected by operation and lymph node metastasis status in the multivariate analysis. Therefore, we concluded that these parameters derived from the peripheral blood were not suitable predictors of disease recurrence. Further studies with larger sample sizes are needed to verify the effects of inflammatory indices on recurrence.

As a retrospective cohort study, our analysis has inherent limitations. While univariate analysis identified SII, LMR, PLR, SIRI, and PIV as predictors of poor overall survival, these indices lost significance in multivariate models, likely due to collinearity with clinical variables or insufficient statistical power from the limited sample size. The retrospective design also introduces potential selection bias and restricts control over confounding factors. We explicitly state that while treatment heterogeneity exists, our statistical analysis did not find it to be a significant confounder in this specific study, thereby strengthening the independent prognostic value of the inflammatory biomarkers we identified. Finally, although lymph node metastasis is a well-established negative prognostic factor, NLR retained its independent prognostic significance even after the exclusion of node-positive (N+) patients, thereby further corroborating our conclusions. Nonetheless, the relatively small number of N+ patients (n=13) limits a more granular analysis of the interaction between inflammatory biomarkers and nodal disease burden, which should be explored in larger, future studies. Furthermore, the lack of standardized cutoff values for inflammatory markers (NLR, SIRI) across studies complicates clinical translation, as thresholds often vary between populations.

To address these limitations, future multicenter, prospective studies with larger cohorts are essential to validate the prognostic utility of PBIBs and establish universal cutoff criteria. Integrating PBIBs with tumor-specific molecular profiling (e.g., PD-L1 status, TP53 mutations) or radiomic features could enhance risk stratification models by improving sensitivity and specificity. Mechanistic investigations should focus on longitudinal monitoring of PBIBs during treatment to clarify their role in predicting therapeutic resistance, while experimental models (e.g., *in vitro* or animal studies) could explore interventions targeting inflammation-immune crosstalk, such as TGF-β or IL-6/STAT3 pathway inhibition, to improve clinical outcomes in lip cancer.

## 5 Conclusions

In the present study, the inflammation index demonstrated predictive power for unfavourable prognosis. A significant association was observed between an increased NLR and increased mortality in patients with lip cancer. The clinical utility of NLR lies in its cost-effectiveness, routine accessibility, and reproducibility, making it a practical tool for risk stratification in resource-limited settings. While other peripheral blood inflammatory biomarkers (SII, PLR, LMR, SIRI, PIV, PNI) demonstrated prognostic potential in univariate analyses, their predictive strength requires validation in larger prospective cohorts. These findings advocate for the integration of NLR into standard prognostic workflows for lip cancer, while underscoring the need for multicenter studies to harmonize cutoff values and explore combinatorial models incorporating molecular biomarkers to refine precision oncology strategies.

## Data Availability

The raw data supporting the conclusions of this article will be made available by the authors, without undue reservation.
